# Crucial roles of XCR1-expressing dendritic cells and the XCR1-XCL1 chemokine axis in intestinal immune homeostasis

**DOI:** 10.1038/srep23505

**Published:** 2016-03-23

**Authors:** Tomokazu Ohta, Masanaka Sugiyama, Hiroaki Hemmi, Chihiro Yamazaki, Soichiro Okura, Izumi Sasaki, Yuri Fukuda, Takashi Orimo, Ken J. Ishii, Katsuaki Hoshino, Florent Ginhoux, Tsuneyasu Kaisho

**Affiliations:** 1 Laboratory for Immune Regulation, World Premier International Research Center Initiative, Immunology Frontier Research Center, Osaka University, Suita, Osaka 565-0871, Japan; 2 Department of Immunology, Institute of Advanced Medicine, Wakayama Medical University, Wakayama, Wakayama 641-8509, Japan; 3 Laboratory of Immune Regulation, Department of Microbiology and Immunology, Graduate School of Medicine, Osaka University, Suita, Osaka 565-0871, Japan; 4 Laboratory for Inflammatory Regulation, RIKEN Center for Integrative Medical Science (IMS-RCAI), Yokohama, Kanagawa 230-0045, Japan; 5 Laboratory for Host Defence, RIKEN Center for Integrative Medical Science (IMS-RCAI), Yokohama, Kanagawa 230-0045, Japan; 6 Department of Immunology, Graduate School of Medicine, Dentistry, and Pharmaceutical Sciences, Okayama University, Okayama, Okayama 700-8558, Japan; 7 Laboratory of Adjuvant Innovation, National Institutes of Biomedical Innovation, Health and Nutrition, Ibaraki, Osaka 567-0085, Japan; 8 Laboratory of Vaccine Science, World Premier International Research Center Initiative, Immunology Frontier Research Center, Osaka University, Suita, Osaka 565-0871, Japan; 9 Department of Immunology, Faculty of Medicine, Kagawa University, Miki, Kagawa 761-0793, Japan; 10 Singapore Immunology Network (SIgN), Agency for Science, Technology and Research (A*STAR), 138648, Singapore

## Abstract

Intestinal immune homeostasis requires dynamic crosstalk between innate and adaptive immune cells. Dendritic cells (DCs) exist as multiple phenotypically and functionally distinct sub-populations within tissues, where they initiate immune responses and promote homeostasis. In the gut, there exists a minor DC subset defined as CD103^+^CD11b^−^ that also expresses the chemokine receptor XCR1. In other tissues, XCR1^+^ DCs cross-present antigen and contribute to immunity against viruses and cancer, however the roles of XCR1^+^ DCs and XCR1 in the intestine are unknown. We showed that mice lacking XCR1^+^ DCs are specifically deficient in intraepithelial and lamina propria (LP) T cell populations, with remaining T cells exhibiting an atypical phenotype and being prone to death, and are also more susceptible to chemically-induced colitis. Mice deficient in either XCR1 or its ligand, XCL1, similarly possess diminished intestinal T cell populations, and an accumulation of XCR1^+^ DCs in the gut. Combined with transcriptome and surface marker expression analysis, these observations lead us to hypothesise that T cell-derived XCL1 facilitates intestinal XCR1^+^ DC activation and migration, and that XCR1^+^ DCs in turn provide support for T cell survival and function. Thus XCR1^+^ DCs and the XCR1/XCL1 chemokine axis have previously-unappreciated roles in intestinal immune homeostasis.

Intestinal immune homeostasis is regulated by a variety of innate and adaptive immune cells^1^, including dendritic cells (DCs)^2^,^3^. The DC population is heterogeneous, consisting of several subsets with specific functions, distinguished by characteristic patterns of surface marker expression^4^,^5^. In mice, CD103^+^CD11b^−^ and CD103^−^CD11b^+^ DC subsets are present in the spleen and lymph nodes (LNs), while in the lamina propria (LP) of the intestine, an additional CD103^+^CD11b^+^ subset exists^4^,^5^. The roles of the various intestinal DC subsets are variously well-studied: CD103^+^CD11b^+^ DCs are the most abundant and are involved in generating Th17 and regulatory T (Treg) cells^6^,^7^,^8^,^9^, and anti-fungal immunity^10^; CD103^−^CD11b^+^ DCs are related to macrophages^2^ and play an immunoregulatory role via the secretion of IL-10 and TGF-β1^11^,^12^; while in contrast, little is known of the role of CD103^+^CD11b^−^ DCs in this site. CD103^+^CD11b^−^ DCs also express the T cell co-receptor CD8α and the chemokine receptor XCR1 (lymphotactin receptor/G-protein-coupled receptor 5), and represent approximately 5% of murine intestinal DCs^13^,^14^,^15^,^16^. This subset (hereafter “XCR1^+^ DCs”) also exists in other tissues including spleen, LNs and skin, where it specializes in the uptake of dead cells and cross-presentation of antigen to CD8^+^ T cells^17^, which is important for protection against viruses, bacteria and parasites, and for anti-tumour immunity^14^,^16^,^18^,^19^,^20^,^21^. XCR1^+^ DCs are also present in other mammalian species^22^,^23^; in humans, XCR1 is expressed on a DC subset that is present in various tissues, including skin and blood, and possesses high cross-presenting activity^24^. How the XCR1^+^ DC subset functions in either the murine or human intestine is currently unknown.

The ligand for XCR1 is XCL1 in mice and XCL1 and XCL2 in humans^25^. In both species, XCL1 is produced mainly by natural killer (NK) and activated CD8^+^ T cells^15^,^16^,^17^,^18^,^22^,^23^. Mice lacking XCR1 or XCL1 show diminished CD8^+^ T cell responses against the antigens cross-presented by CD103^+^CD11b^−^ DCs^15^; XCL1 is also involved in regulating medullary accumulation of thymic XCR1^+^ DCs, and thymic generation of naturally-occurring regulatory T (Treg) cells^26^. Thus, the XCR1-XCL1 axis has the potential to modulate both the localization and function of T cells and DCs, though the extent to which this is relevant outside of the thymic environment is not yet clear.

In order to clarify the functions of XCR1^+^ DCs, we generated and analysed mutant mice in which these cells are constitutively ablated. These mice possessed significantly fewer intestinal T cells than their wildtype counterparts, and the remaining T cells exhibited an atypical phenotype. Consistent with the regulatory roles of intestinal T cells, XCR1^+^ DC-deficient mice showed exaggerated manifestations during chemically-induced colitis. Alongside, in mice lacking either XCR1 or XCL1, a similar decrease in T cell populations was seen, accompanied by an accumulation of CD103^+^CD11b^−^ DCs in the intestine. Thus, we have identified novel regulatory roles of XCR1^+^ DC and the XCR1-XCL1 axis in maintaining intestinal immune homeostasis, and have proposed a hypothetical model on which to base future studies to define the underlying mechanisms.

## Results

### Intestinal T cell populations are specifically decreased in mice lacking XCR1^+^ DCs

We exploited the specific expression of XCR1 on this DC subset to generate a mouse model in which CD103^+^CD11b^−^ XCR1^+^ DCs are constitutively ablated as a result of diphtheria toxin A subunit (DTA) expression (XCR1-DTA mice)(Supplementary Fig. 1A,B). We first confirmed the ablation of CD103^+^CD11b^−^ DCs in the LP, mesenteric lymph nodes (MLNs) and spleens of XCR1-DTA mice, and the retention of the other DC subsets across these tissues (Supplementary Fig. 1C). In the spleen, CD103^+^CD11b^−^ DCs were more severely ablated than CD8α^+^CD11b^−^ DCs, which is consistent with the finding that CD8α^+^CD11b^−^ DC populations contain more XCR1^−^ cells than CD103^+^CD11b^−^ DC populations^13^,^14^ (Supplementary Fig. 1D).

**Figure 1:**
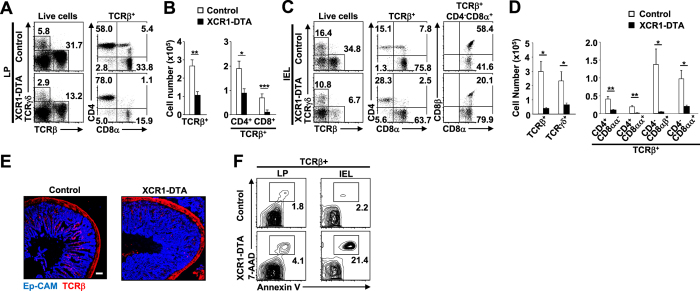
Intestinal T cell populations are decreased in mice lacking XCR1^+^ DCs. (**A–D**) Percentages (**A**,**C**) and total numbers (**B**,**D**) of T lineage cells of LP (**A**,**B**) and IELs (**C**,**D**) from control (*Xcr1*^*+/cre*^) and XCR1-DTA mice. Dead cells and doublets were eliminated by FSC/SSC gating and LIVE/DEAD staining, with live cells subjected to further gating as indicated. (**E**) Immunofluorescent images of intestinal sections from control and XCR1-DTA mice labelled with anti-TCRβ and anti-Ep-CAM Abs. Scale bars, 500 μm. Means ± s.e.m. of five mice are indicated (**B**,**D**). (**F**) AnnexinV and 7-AAD staining of LP and intraepithelial TCRαβ^+^ cells, in control and XCR1-DTA mice. Percentage of double-positive cells is indicated. Results are representative of five (**A**-**D**) or two (**E**,**F**) independent experiments. (*P < 0.05; **P < 0.01; ***P < 0.001, Student’s *t* test).

We next asked whether T cell populations were affected by the absence of XCR1^+^ DCs. In thymus, spleen and MLNs, the size and composition of the T cell populations were comparable between control and XCR1-DTA mice (Supplementary Fig. 2A,B). Normally the intestine contains several distinct lymphocyte populations: within the LP, conventional TCRαβ^+^ CD4^+^ and CD8^+^ T cells predominate, while the intraepithelial lymphocyte (IEL) population additionally includes TCRγδ^+^ T cells and an atypical TCRαβ^+^ subset that expresses CD8αα instead of CD8αβ^27^. In the LP of XCR1-DTA mice there were approximately two-thirds fewer TCRαβ^+^ T cells than in control mice, with significant decreases across both CD4^+^ and CD8^+^ subpopulations (Fig. 1A,B); all IEL subsets were also significantly reduced compared to controls (Fig. 1C,D). Moreover, taking into consideration the lower T cell numbers, there was a further relative decrease in the more mature CD4^+^CD8αα^+^ IEL subset, accompanied by a relative accumulation of the less mature CD4^+^CD8^−^ IELs in XCR1-DTA mice (Fig. 1C). The relative scarcity of TCRβ- expressing cells was similarly evident histologically (Fig. 1E). Thus, XCR1^+^ DCs are critical for keeping both intestinal LP T cells and IEL populations.

### Intestinal T cells are more prone to death in XCR1-DTA mice

The low numbers of intestinal T cells in XCR1-DTA mice might have been due to decreased proliferation or increased cell death. The proliferation marker Ki67 was expressed at a comparable frequency in LP T cell and IEL sub-populations from XCR1-DTA and control mice (Supplementary Fig. 3A,B). However, there were markedly more dead and/or dying intestinal TCRαβ^+^ T cells in mice lacking XCR1^+^ DCs, in particular in the IEL compartment, as assessed by frequency of Annexin V and 7-amino-actinomycin D (7-AAD) staining (Fig. 1F). The findings indicate that the T cells present in XCR1-DTA mice were not defective in proliferation, but were more prone to undergo cell death, likely contributing to the T cell deficiency observed in these mice. Thus, the absence of XCR1^+^ DCs compromises the survival of intestinal T cell populations.

### LP T cells exhibit an abnormal phenotype in XCR1-DTA mice

**Figure 2:**
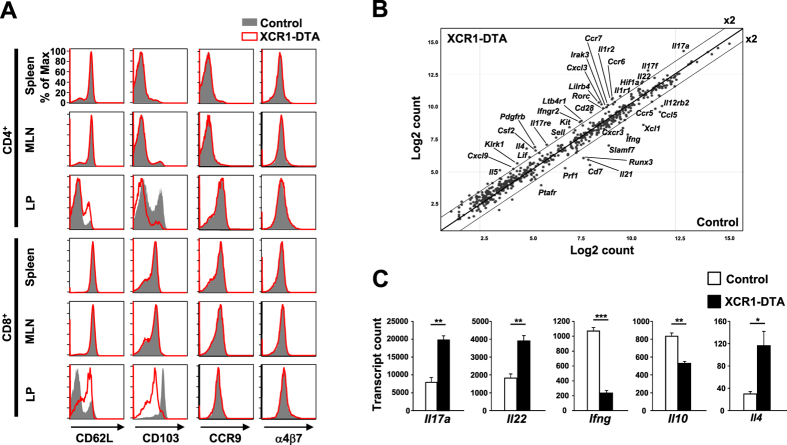
Intestinal T cells in mice lacking XCR1^+^ DCs exhibit an aberrant phenotype. (**A**) Intensity of expression of CD62L, CD103, CCR9 and α4β7 integrin on CD4^+^ and CD8^+^ T cells in spleen, MLNs and LP from control (*Xcr1*^*+/cre*^, shaded histograms) and XCR1-DTA (open histograms with red lines) mice. Results are representative of four independent experiments. (**B**,**C**) Nanostring gene expression analysis of purified LP CD4^+^ T cells from control and XCR1-DTA mice. Scatter plot (**B**) shows means of normalized log intensities of individual probes with lines indicating the 2-fold difference threshold. Expression profiles (**C**) of indicated genes are shown as means ± s.e.m. of three independent experiments. Cells from three or four mice were pooled for each experiment.

We next compared the phenotype of the lymphoid tissue T cells from control and XCR1-DTA mice. In control mice, LP TCRαβ^+^ T cells expressed lower levels of the cell adhesion molecule CD62L and higher levels of the integrin CD103, compared with splenic and MLN TCRαβ^+^ T cells (Fig. 2A, shaded histograms). However, LP TCRαβ^+^ T cells from XCR1-DTA mice showed defects in this intestinal T cell phenotype (Fig. 2A). Expression of the gut-homing receptors α4β7 integrin and CCR9^28^,^29^ was comparable between control and XCR1-DTA mice (Fig. 2A).

We then asked whether LP T cells from XCR1-DTA and control mice further differed at the transcriptional level. LP CD4^+^ T cells from mice lacking XCR1^+^ DCs contained significantly more transcripts for *Il17a, Il22* and *Il4* and significantly fewer transcripts for *Ifng* and *Il10* than those from control mice (Fig. 2B,C), suggesting polarization towards a Th2/17 profile, rather than the Th1 profile evident in control animals. Furthermore, it is notable that *Runx3* expression in CD4^+^ T cells from XCR1-DTA mice was decreased compared with that of CD4^+^ T cells from control mice. A transcription factor, RUNX3, is required for transition of CD4^+^CD8^−^ to CD4^+^CD8αα^+^ T cells^30^,^31^. Therefore, decrease of *Runx3* expression might explain the observed block in the generation of CD4^+^CD8αα^+^ from CD4^+^CD8^−^ T cells in XCR1-DTA mice (Fig. 1C). Thus, gene expression profile analysis revealed that LP T cell surface phenotype and gene expression profile is profoundly altered in mice lacking XCR1^+^ DCs.

Given the known role of XCL1 in the thymic generation of natural Treg cells^26^ we next investigated whether the intestinal Treg population was affected in XCR1-DTA mice. The proportion of Treg cells, defined as CD25^+^Foxp3^+^, among CD4^+^ T cells, and the expression of the natural Treg marker, Nrp1^32^, were comparable between control and XCR1-DTA mice (Supplementary Fig. 4A–C). In line with the overall CD4^+^ T cell decrease in XCR1-DTA mice, the number of LP Tregs was approximately half that of control mice (Supplementary Fig. 4C), but the remaining cells existed at the expected frequency and exhibited a normal phenotype. Therefore, in contrast to other LP T cell populations, the generation and maintenance of Tregs is not specifically affected in mice lacking XCR1^+^ DCs.

### XCR1-DTA mice are more susceptible to DSS-induced colitis

**Figure 3:**
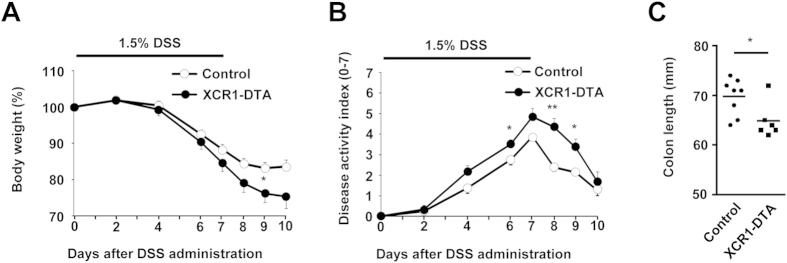
XCR1-DTA mice are more susceptible to DSS-induced colitis. (**A**,**B**) Body weight changes (**A**) and disease activity index (**B**) after DSS exposure of control (open circles) and XCR1-DTA (filled circles) mice. Mice were given 1.5% DSS in drinking water for 7 days and clinically monitored. Means ± s.e.m. of control (n=8) and XCR1-DTA (n=6) mice are indicated. (**C**) Colon length after 10 days of DSS treatment. Means are shown as bars. Results are representative of three (**A-C**) independent experiments. (*P < 0.05; **P < 0.01, Student’s *t* test).

While mice lacking all CD11c^+^ DCs exhibit aberrant myeloid cell proliferation and autoimmunity^33^, the absence of CD103^+^CD11b^−^ DCs alone does not induce any overt signs of dysregulated immunity under steady-state conditions^34^. However, during dextran sodium sulphate (DSS) -induced colitis, while mice lacking CD103^+^CD11b^−^ DCs through deficiency in the transcription factor BATF3 (Basic leucine zipper transcription factor ATF-like 3) (BATF3) are as susceptible as their wild-type counterparts^34^, induced ablation of CD103^+^CD11b^−^ DCs in Clec9A-diphtheria toxin receptor (DTR) mice resulted in significant exacerbation of colitis^35^. As XCR1-DTA mice also did not show clear signs of dysregulated intestinal immunity/intestinal inflammation in the steady state, we assessed their susceptibility to DSS-induced colitis. After administration of DSS, XCR1-DTA mice lost significantly more weight, achieved significantly higher disease activity scores and underwent significantly more colon shortening compared to control mice (Fig. 3). Thus, while the marked differences in the intestinal immune compartment in XCR1-DTA mice do not result in inflammatory changes under homeostatic conditions, these animals are rendered exquisitely sensitive to induced intestinal inflammation.

### Mice lacking XCR1 or XCL1 exhibit similar defects in intestinal T cell populations as XCR1-DTA mice

**Figure 4:**
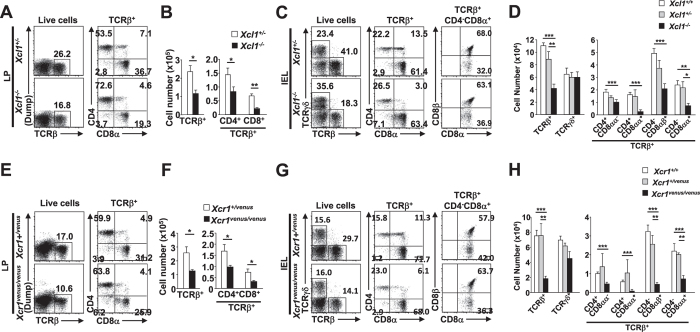
The XCR1-XCL1 axis is involved in maintenance of intestinal T cell populations. (**A–D**) Percentages (**A**,**C**) and total numbers (**B**,**D**) of T lineage cells of LP and IELs from control (*XCL1*^*+/−*^) and XCL1-deficient (*XCL1*^*−/−*^) mice are shown. (**E–H**) Percentages (**E**,**G**) and total numbers (**F**,**H**) of T lineage cells of LP and IELs from control (*XCR1*^*+/venus*^) and XCR1-deficient (*XCL1*^*venus/venus*^) mice are shown. Dead cells and doublets were eliminated by FSC/SSC gating and LIVE/DEAD staining, with live cells subjected to further gating as indicated. Means ± s.e.m. of five mice are shown (**B**,**D**,**F**,**H**). Results are representative of four independent experiments. (*P < 0.05; **P < 0.01; ***P < 0.001, Student’s *t* test).

We then asked whether it was possible that the XCR1-XCL1 axis itself was contributing to the observed defects in mice lacking XCR1^+^ DCs. We first generated XCL1-deficient mice (Supplementary Fig. 5A,B), and found that the size and composition of the T cell population in spleen and MLNs was comparable with control mice (Supplementary Fig. 6A,B). However, similar to XCR1-DTA mice, XCL1-deficient mice possessed significantly fewer TCRαβ^+^ T cells in their LP and intraepithelial compartment (Fig. 4A–D). All the LP and intraepithelial TCRαβ^+^ T cell subsets, but not TCRγδ^+^ T cells, were significantly decreased in XCL1-deficient mice compared to controls. Relative proportions of CD4^+^CD8^−^ and CD4^+^CD8αα^+^ T cells were also increased and decreased, respectively within the IEL population (Fig. 4C), as in XCR1-DTA mice (Fig. 1C).

We then analysed XCR1-deficient mice^16^. These animals also possessed significantly fewer LP and intraepithelial TCRαβ^+^ T cells than controls (Fig. 4E–H), while splenic and MLN T cell populations were comparable between control and mutant mice (Supplementary Fig. 6C,D). Furthermore, all LP and intraepithelial TCRαβ^+^ T cell subsets, but not TCRγδ^+^ T cells, were decreased in XCR1-deficient mice.

In summary, these data show that mice lacking either XCL1 or XCR1 exhibit similar specific defects in intestinal T cell populations as XCR1-DC-deficient mice. Therefore it is possible that the XCR1-XCL1 axis itself is directly involved in intestinal T cell homeostasis, via a mechanism that might either involve interaction with XCR1^+^ DCs or be independent of this pathway.

### XCR1 deficiency alters the gene expression profile of intestinal DCs

**Figure 5:**
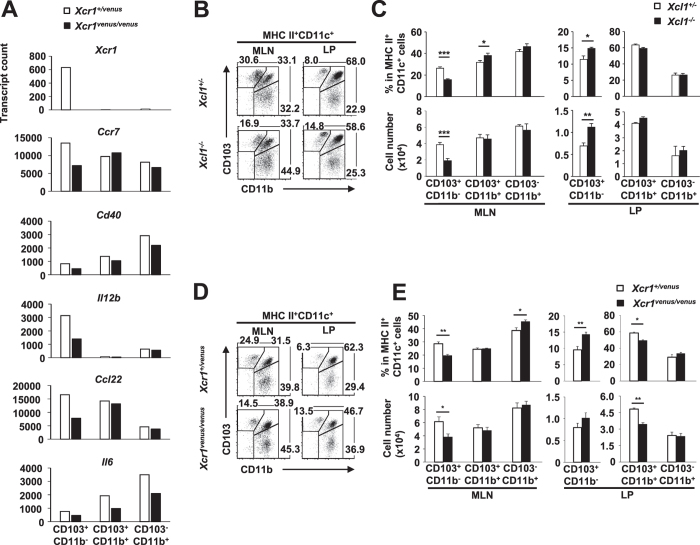
XCR1^+^ DC distribution is affected in the absence of XCR1 or XCL1. (**A**) Nanostring gene expression analysis of sorted LP DC subsets pooled from eight *Xcr1*^*+/venus*^ or eight *Xcr1*^*venus/venus*^ mice. (**B**,**C**) Percentages (**B**) and total numbers (**C**) of MLN and LP DCs from control (*XCL1*^*+/−*^) and XCL1-deficient (*XCL1*^*−/−*^) mice are shown. (**D**,**E**) Percentages (**D**) and total numbers (**E**) of MLN and LP DCs of control (*XCR1*^*+/venus*^) and XCR1-deficient (*XCL1*^*venus/venus*^) mice are shown. Dead cells and doublets were eliminated by FSC/SSC gating and LIVE/DEAD staining, with live cells subjected to further gating as indicated. Results are representative of four (**C**,**E**) independent experiments. Means ± s.e.m. of five mice are indicated (**C**,**E**). (*P < 0.05; **P < 0.01; ***P < 0.001, Student’s *t* test).

To further dissect the direct effects of the XCR1-XCL1 axis on DCs in the gut, we compared the gene expression profiles of LP DC subsets from control and XCR1-deficient mice. The mutant CD103^+^CD11b^−^ DCs contained significantly fewer transcripts for 62 of the 547 immune-related genes measured than did control CD103^+^CD11b^−^ DCs. The most highly differentially-expressed genes within this subset were *Xcr1, Ccr7, Cd40, Il12b, Ccl22* and *Il6* (Fig. 5A). *Ccr7* encodes the chemokine receptor, CCR7, which is required for DCs to migrate from the peripheral tissues to the draining LNs^36^; thus low expression of *Ccr7* might impede emigration from the LP and thus result in abnormal patterns of DC distribution. To assess the biological significance of this finding, we measured the relative abundance of CD103^+^CD11b^−^ DCs in the LP and MLNs of mice lacking either XCL1 or XCR1, and in control animals. As would be expected if DCs expressed insufficient CCR7 for effective migration to the LN, in both XCL1- and XCR1- deficient mice, the CD103^+^CD11b^−^ DC population was significantly smaller in the MLN and significantly larger in the LP, compared to control mice (Fig. 5B–E). Meanwhile, the composition of the splenic DC compartment was unaffected by the absence of XCL1 or XCR1 (Supplementary Fig. 7A–D). Thus it appears that the XCR1-XCL1 axis is required for optimal gene expression to support normal migration of intestinal XCR1^+^ DCs to the MLN.

**Figure 6:**
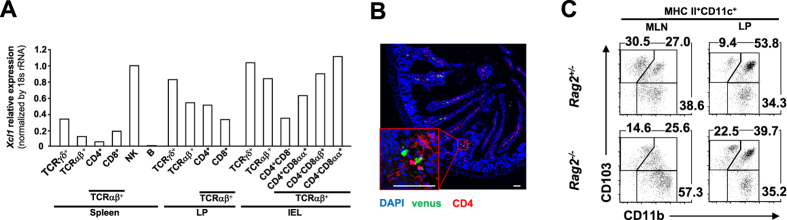
Intestinal CD4 T cells are closely associated with XCR1^+^ DCs *in vivo*. (**A**) *Xcl1* expression in sorted T cells from spleen and LP, IELs, and splenic B (CD3ε^−^B220^+^) and NK (CD3ε^−^CD49b^+^) cells pooled from eight wildtype mice, determined by quantitative real-time PCR. (**B**) Immunofluorescence imaging of intestinal sections from *Xcr1*^*+/venus*^ mice labelled with anti-CD4 Ab and venus. Scale bars, 60 μm. (**C**) Percentages of MLN and LP DC subsets in *Rag2*^*+/−*^ and *Rag2*^*−/−*^ mice. Dead cells and doublets were eliminated by FSC/SSC gating and LIVE/DEAD staining, with live cells subjected to further gating as indicated. Results are representative of two independent experiments.

As XCL1 is known to be produced by NK and CD8 T cells in other tissues^15^,^16^,^17^,^18^,^22^,^23^, and given the observed importance of XCL1 for optimal intestinal DC function, we then asked which cells within the intestinal compartment express *Xcl1* and might therefore act as a source of the chemokine *in vivo*. Compared with splenic T cells, LP and intraepithelial T cell subsets from wildtype mice showed high expression of *Xcl1* (Fig. 6A). Interestingly, we saw that *Xcl1* expression in LP T cells of XCR1-DTA mice was decreased to approximately 20% of that in control mice (Fig. 2B), which might indicate that the high expression of *Xcl1* in intestinal T cells is supported by the presence of XCR1^+^ DC. Immunofluorescence imaging of intestinal sections confirmed that XCR1^+^ DCs and T cells are closely associated *in vivo* (Fig. 6B). Furthermore, in similar to XCL1- or XCR1-deficient mice, RAG2-deficient mice, which lack any mature lymphocytes, showed the increase and decrease of CD103^+^CD11b^−^ DCs in the LP and MLN, respectively (Fig. 6C). Taken together, these data suggest the possibility that intestinal T cells might provide XCR1^+^ DCs with XCL1 while closely associated in the gut.

## Discussion

Here we have generated and analysed XCR1-DTA mice which lack the CD103^+^CD11b^−^XCR1^+^ DC subset with the aim of understanding the role of this DC subset. While these mice did not exhibit any overt signs of pathology as a result of the absence of XCR1^+^ DCs, all LP T cell and IEL populations were specifically and significantly decreased, while the abundance of T lineage cells in thymus, spleen and MLNs was not affected. Thus the presence of XCR1^+^ DCs is necessary for normal numbers of T cells in the intestinal LP and IEL compartments.

In contrast to the absence of pathology in the steady-state, we found that the defects induced by the absence of XCR1^+^ DCs rendered XCR1-DTA mice significantly more susceptible to induced colitis compared with control mice. While the pathology of DSS colitis does not require the presence of T cells^37^, the condition is exaggerated in various mutant mice in which IELs, or in particular CD4^+^CD8αα^+^ T cells, are depleted^27^,^38^,^39^,^40^,^41^,^42^. Exacerbation of colitis in XCR1-DTA mice, with their profoundly diminished intestinal T cell populations, is consistent with these reports. Thus, while the absence of XCR1^+^ DCs does not cause inflammation per se, the associated quantitative and/or qualitative changes in the intestinal T cell compartment may reduce the ability of XCR1-DTA mice to control the response to DSS-induced gut inflammation.

Similar to our findings, mice lacking CD103^+^CD11b^−^ DCs do not exhibit signs of spontaneous inflammation in the steady state^34^,^35^, but their susceptibility to DSS-induced colitis seems to vary depending on additional factors: models using deficiency in the transcription factor BATF3 to induce CD103^+^CD11b^−^ DC deficiency are as susceptible as their wild-type counterparts^34^, while employing diphtheria-toxin-mediated ablation of CD103^+^CD11b^−^ DCs in Clec9A-DTR mice renders them significantly more sensitive to induced colitis^35^. Alongside the difference in DC ablation method, susceptibility to DSS-induced colitis can be affected by differences in the commensal gut microbiota, mouse genetic background and source and dose of the DSS used. For example, Edelson *et al*. used 129S6/SvEv mice with 5% DSS from TDB Consultancy^34^, whereas we used C57BL/6 mice with 1.5% DSS from MP Biomedicals. Furthermore, it cannot be formally excluded that CD103^+^CD11b^−^ DCs are compensatory generated during the course of DSS-induced colitis in BATF3-deficient mice^43^. Meanwhile, our results are consistent with enhanced manifestations of DSS-induced colitis in Clec9A-DTR mice, in which CD103^+^CD11b^−^ DCs are inducibly ablated^35^. Muzaki *et al*. used BALB/c Clec9A-DTR mice treated with 2% DSS from MP Biomedicals and showed that CD103^+^CD11b^−^ DCs were critical for anti-inflammatory responses, dependent on T cell-derived IFN-γ; although intestinal T cell populations were not investigated in their study, we also observed decreased *Ifng* expression in the remaining LP CD4^+^ T cells from XCR1-DTA mice prior to the induction of colitis (Fig. 2C).

In addition to the depletion of T cell populations and an inability to restrain induced inflammation, we detected further abnormalities in the intestinal T cell compartment of mice lacking XCR1^+^ DCs. Intestinal T cells normally express lower levels of CD62L and higher levels of CD103 than T cells in other tissues, however LP T cells from XCR1-DTA mice showed defects in this typical intestinal phenotype. Decreased expression of CD62L is indicative of the constitutive activation of intestinal T cells, most likely by antigens from the intestinal lumen, such as those of the commensal bacteria. Indeed, germ-free or antibiotic-treated mice also show decreased populations of intestinal T cells including IELs^44^,^45^. Therefore, the failure to downregulate CD62L of LP T cells from XCR1-DTA mice indicates a deficiency somewhere along the pathway leading to activation of these T cells, or in their ability to respond to activating stimuli. It is unknown whether XCR1^+^ DCs directly incorporate and present antigens in the gut, though there is evidence that CX3CR1^+^ macrophages can capture and transfer antigens to CD103^+^ DCs^46^. It will be intriguing to investigate further how the absence of XCR1^+^ DCs is leading to the emergence of a diminished T cell population with atypical activation characteristics.

The upregulated expression of CD103 by intestinal T cells is driven by the local cytokine milieu, and particularly by TGF-β^47^. The absence of either CD103 or functional TGF-β signalling in T cells results in impaired development of intestinal T cell populations^48^,^49^, as seen in XCR1-DTA mice. TGF-β is normally produced by intestinal DCs, including XCR1^+^ DCs^11^, therefore we speculate that the lack of effective CD103 upregulation by intestinal T cells in XCR1-DTA mice might be caused by an absence of XCR1^+^ DC-derived TGF-β, which could also contribute to the decrease in T cell populations.

Intestinal T cells in XCR1-DTA mice had poor survival capacity and a tendency to express genes related to Th2 or Th17 differentiation, rather than Th1. It is unclear how the absence of XCR1^+^ DC is linked to this defect, however BATF3-dependent DCs are known to be involved in Th1 cell differentiation^20^, so it is possible that XCR1^+^ DCs might also drive T cell polarization. Meanwhile, Treg cells were numerically reduced in line with the overall decrease in T cell populations, but we did not detect any abnormalities in their phenotype in XCR1-DTA mice. Similarly, BATF3-deficient mice maintained a normal population of Treg cells in their LP and MLNs^34^.

Absence of XCR1^+^ DCs also led to further relative decrease in CD4^+^CD8αα^+^ IELs, accompanied by a relative accumulation of the CD4^+^CD8^−^ T cells in XCR1-DTA mice. The generation requires certain MHC class II-expressing antigen presenting cells and *Runx3* expression in CD4^+^CD8^−^ T cells^30^,^31^. Because *Runx3* expression in CD4^+^CD8^−^ T cells was decreased in XCR1-DTA mice, we assume that XCR1^+^ DCs are responsible for the generation of CD4^+^CD8αα^+^ T cells by keeping *Runx3* expression levels. *Runx3* expression is driven by TGF-β and retinoic acids, which are generated by aldehyde dehydrogenase from vitamin A^30^,^31^. XCR1^+^ DCs express both *Tgf-b1* and *aldh1a2*^11^. Although further studies are necessary to clarify whether the decrease of CD4^+^CD8αα^+^ T cells in XCR1-DTA mice is caused by developmental block and/or impaired expansion, it is possible that XCR1^+^ DCs are required for the generation of CD4^+^CD8αα^+^ T cells by providing TGF-β and/or retinoic acids.

While the removal of XCR1^+^ DCs clearly induces profound changes in the intestinal immune compartment, the question remains: to what extent is the XCR1-XCL1 axis involved, or are the observed effects more likely to be due to other, XCR1-XCL1-independent, effects? While this study was not designed to answer this question specifically, we found that mice lacking XCR1 or XCL1 recapitulated important features of the T cell insufficiencies and imbalances present in XCR1-DTA mice. Moreover, intestinal T cells from wild type mice abundantly expressed *Xcl1* and were observed in close association with XCR1^+^ DCs in tissue sections, while LP T cells from XCR1-DTA mice expressed significantly lower levels of *Xcl1* than those from control mice (Fig. 2B). Thus it is possible that intestinal T cells provide XCL1 to local XCR1^+^ DCs. We also noted that in XCR1- and XCL1-deficient mice, CD103^+^CD11b^−^ DCs accumulated in the LP, and were reduced in number in the MLN: in XCR1-deficient mice at least, this could be due to the lower than normal level of *Ccr7* expression in CD103^+^CD11b^−^ DCs, as CCR7 is required for intestinal DCs to migrate to the MLN^50^.

**Figure 7:**
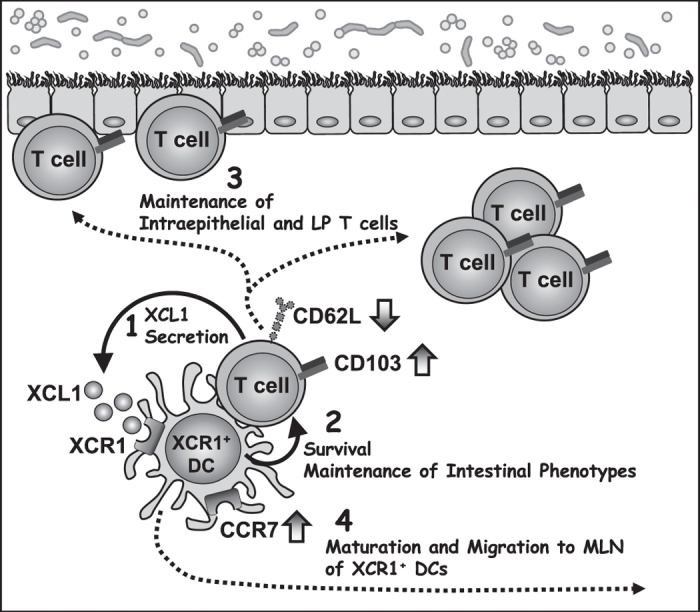
Hypothetical model of the possible crosstalk between XCR1^+^ DCs and intestinal T cells. Activated intestinal T cells produce XCL1, which attracts nearby XCR1^+^ DC; the ensuing DC-T cell crosstalk supports T cell survival, promotes upregulation of CD103 expression with downregulation of CD62L expression, and leads to maintenance of intraepithelial and LP T cells; continuing XCL1 expression by the T cells in turn enables DC maturation, including upregulated CCR7 expression to enable migration from the LP to the MLN.

Taken together, our findings lead us to propose a hypothetical model of XCR1-DC-T cell crosstalk in the intestine as a basis for further study and discussion (Fig. 7): activated intestinal T cells produce XCL1, which specifically attracts nearby XCR1^+^ DC; the ensuing DC-T cell crosstalk supports T cell survival, promotes the typical CD103^high^/CD62L^low^ intestinal phenotype, and leads to maintenance of intraepithelial and LP T cells; continuing XCL1 expression by the T cells in turn enables DC maturation, and in particular CCR7 expression necessary for migration from the LP to the MLN.

In conclusion, our findings highlight the potential significance of the XCL1-XCR1 axis, and in particular the interaction of XCR1^+^ DCs and the intestinal T cell compartment, in maintaining gut homeostasis and ensuring appropriate control of inflammation. This both advances our understanding of the fundamental workings of the intestinal immune system and identifies the XCR1-XCL1 axis as a novel potential therapeutic target for the treatment of human intestinal immune disorders. Further work to identify the key molecular mediators and underlying mechanisms will be necessary to fully understand this intriguing system.

## Methods

### Mice

C57BL/6J mice were purchased from CLEA Japan. All mice were bred and maintained in the Animal Facility of RIKEN Research Institute for Allergy and Immunology (Yokohama, Japan), Animal Resource Center for Infectious Diseases, Research Institute for Microbial Diseases and Immunology Frontier Research Center, Osaka University (Suita, Japan) and Institute for Animal Experimentation, Wakayama Medical University (Wakayama, Japan) under specific pathogen-free conditions and were used at 7–14 weeks of age according to the institutional guidelines of RIKEN Institute, Osaka University and Wakayama Medical University. All animal experiments were approved by the animal research committees and were carried out in accordance with approved guidelines of the animal care committees of of RIKEN Yokohama Research Institute, Osaka University and Wakayama Medical University.

### Generation of mutant mice

XCR1-DTA mice were generated as follows: first, the entire murine XCR1 coding sequence was replaced with a gene encoding the Cre recombinase to generate mice in which Cre recombinase is expressed in XCR1-expressing cells (Supplementary Fig. 1A). The Cre recombinase gene was derived from pPGK-Cre-bpA (kindly provided by Dr. Klaus Rajewsky, Max-Delbrück-Center for Molecular Medicine, Berlin, Germany) and carried a polyadenylation signal derived from the bovine growth hormone gene (*bGHpA*). A neomycin-resistance gene driven by the MC1 promoter and flanked by yeast FRT sequences was used as a selection marker. A herpes simplex virus thymidine kinase gene (HSV-TK) was inserted for negative selection. The C57BL/6-derived embryonic stem (ES) cell line, Bruce4 (kindly provided by Drs. Colin L. Stewart, Institute of Medical Biology, Singapore, through Masaki Hikida, Kyoto University, Japan), was transfected with the linearized targeting vector by electroporation and selected with G418 (Nacalai Tesque) and ganciclovir (Mitsubishi Tanabe Pharma). Doubly-resistant clones were screened for homologous recombination by PCR and verified by Southern blot analysis. Germline-transmitting chimeras were generated by injection of targeted ES clones into blastocysts from BALB/c mice which were bred with C57BL/6J mice. *Xcr1*^*+/cre*^ mice were further backcrossed with C57BL/6J mice for 2–6 generations. *Xcr1*^*+/cre*^ mice were crossed with R26:lacZbpA^flox^DTA mice (kindly provided by Dr. Dieter Riethmacher, University of Southampton, UK, through Dr. Shigekazu Nagata, Kyoto University, Japan) carrying the DTA gene, which is designed to be expressed specifically in the cells or tissues expressing Cre recombinase under the control of the ubiquitously-active Rosa26 promoter^51^. This crossing generated the XCR1-DTA mice, in which XCR1^+^ cells are ablated by DTA expression. *Xcr1*^*+/cre*^ littermates were used as controls.

*Xcr1*^*+/venus*^ mice were generated by knocking a gene encoding a fluorescent protein, venus, into the *Xcr1* locus^16^. *Xcr1*^*venus/venus*^ mice were therefore rendered XCR1-deficient.

To generate XCL1-deficient mice, the targeting vector was designed to replace the murine XCL1 coding sequences with a neomycin resistance gene driven by the MC1 promoter, flanked by the yeast FRT sequence (Supplementary Fig. 5A). Bacteriophage P1 loxP sequences and the DTA gene were inserted for conditional ablation and negative selection, respectively. The targeted ES cells were generated as described above, but were selected with G418 alone. The neomycin resistance gene was removed by crossing with CAG-*cre* transgenic mice (kindly provided by Jun-ichi Miyazaki, Osaka University, Osaka, Japan)^52^, and the resultant *Xcl1*^*+/−*^ mice were then backcrossed with C57BL/6J mice for 4 generations. The resulting *Xcl1*^*−/−*^ mice were thus XCL1 deficient.

### Cell preparations

Thymocyte and splenocyte suspensions were prepared by grinding the appropriate organs through mesh filters. MLNs were digested with 400 units/ml collagenase III (Worthington) and 100 μg/ml deoxyribonuclease (DNase) I (Sigma-Aldrich) at 37 °C for 20 min. Intestinal immune cells were prepared from the small intestine as follows: fat and Peyer’s patches were dissected away before the small intestine was opened longitudinally and stirred in RPMI containing 2% FCS, 2 mM EDTA for 20 min at 37 °C. IELs were isolated from the resulting supernatant using a 40–75% Percoll density gradient (GE Healthcare) and by collecting the cells that layered between the 40 and 75% fractions. Following supernatant collection the intestinal tissue was stirred for an additional 20 min at 37 °C in RPMI containing 2% FCS, before mincing and further stirring in 400 units/ml of collagenese D (Roche Diagnostics) and 10 μg/ml DNase I for 20 min at 37 °C. Floating cells were collected and the collagenase digestion was repeated twice more. The pooled cell suspensions were then centrifuged on a 40–70% Percoll density gradient, and the cells that layered between the 40–75% fractions were collected as LP cells.

### Flow cytometric analysis

Single cell suspensions were incubated with anti-CD16/32 Ab (TONBO Biosciences) to block non-specific binding of Abs. The cells were then labelled with fluorochrome-conjugated Abs and/or biotinylated Abs against murine CD4 (GK1.5), CD8α (53-6.7), CD8β (H35-17.2), CD11b (M1/70), CD11c (N418), CD25 (PC61), CD62L (MEL-14), CD103 (M290), CCR9 (CW-1.2), α4β7 (DATK32), TCRβ (H57-597), TCRγδ (GL3), I-A/I-E (M5/114.15.2) or Foxp3 (FJK-16s). Biotinylated Abs were visualized via fluorochrome-conjugated streptavidin. Abs and fluorochrome-conjugated streptavidin were purchased from BD Biosciences, eBioscience, BioLegend and TONBO biosciences. Biotinylated anti-Nrp1 Ab and normal goat IgG were purchased from R&D. Foxp3 was detected intracellularly using Foxp3 Staining Buffer Set (eBioscience). Dead cells were excluded using the LIVE/DEAD Fixable Dead Cell Stain Kit (Invitrogen). Cells were analysed on a FACSVerse or FACSAria II (BD Biosciences) and data analysed with FlowJo software (TreeStar).

### Histology

For Fig. 1E, small intestines were embedded in FSC22 frozen section compound (Leica Microsystems). Five μm cryosections were labelled with rat anti-human Ep-CAM (G8.8; BioLegend) Ab and biotinylated armenian hamster anti-TCRβ (H57-597; BioLegend) Ab, and then labelling was visualised with streptavidin-Alexa Fluor 594 (Invitrogen) and Alexa Fluor 647-conjugated anti-rat IgG (H+L) Ab (Invitrogen). For Fig. 6B, small intestines from *Xcr1*^*+/venu*s^ mice were first fixed with 4% paraformaldehyde and then embedded in FSC22 frozen section compound. Five μm cryosections were labelled with rat anti-mouse CD4 Ab (BioLegend) and labelling visualised with goat anti-Rat IgG Ab conjugated-Alexa Fluor 594 (Invitrogen). Sections were examined using the FV10i microscope (Olympus).

### Cell death and proliferation analysis

For analysis of cell death, LP cells and IELs were washed with annexin-binding buffer (10 mM HEPES, 140 mM NaCl, 2.5 mM CaCl2, pH 7.4) and incubated with fluorochrome-conjugated annexin-V and 7-AAD Viablity Staining Solution (eBioscience) for 15 min at room temperature. For analysis of cell proliferation, cells were surface-labelled with the appropriate conjugated Abs, fixed, permeabilized using Foxp3 Staining Buffer Set (eBioscience) and subsequently incubated with anti-Ki67-FITC (B56; BD Biosciences) for 30 min. After washing, cells were analysed on a FACSAria II.

### Gene expression analysis

Gene expression profiles of LP CD4^+^ T cells (Fig. 2B) and DC subsets (Fig. 5A) were analysed with the Mouse Immunology Kit containing 547 immune-related genes and the NanoString nCounter gene expression system (NanoString Technologies, Seattle, WA). Briefly, 10,000 cells of each T cell or DC subset were lysed in RLT buffer (Qiagen, Valencia, CA). The lysates were hybridized for 16 h with the Mouse Immunology Kit and loaded into the nCounter prep station followed by quantification using the nCounter Digital Analyzer (NanoString Technologies). The nCounter data were normalized in two steps: firstly using the positive spiked-in controls provided by the nCounter instrument (NanoString Technologies), and secondly, according to the expression of 14 control genes (*Alas1, Eef1g, G6pdx, Gapdh, Gusb, Hprt, Oaz1, Polr1b, Polr2a, Ppia, Rpl19, Sdha, Tbp* and *Tubb5*).

### Colitis model

Mice were allowed free access to filtered drinking water containing 1.5% DSS (MW = 36,000–50,000; MP Biomedicals) for 1 week. Mice were weighed at indicated days. Severity of colitis was assessed using the disease activity index based on presence of diarrhea and bleeding^53^. At day 10 all mice were euthanized and colons were dissected for measurement of length.

### Quantitative real-time PCR

Total RNAs were obtained using the Sepasol RNA-I Super G (Nacalai tesque), reverse transcribed and analysed by an ABI PRISM 7000 (Applied Biosystems). TaqMan probes (TaqMan Gene Expression Assay; Applied Biosystems) were used for 18S rRNA (internal control). The following SYBR Green primers were used: *Xcl1* (Forward 5′- TTTGTCACCAAACGAGGACTAAA-3′, Reverse 5′-CCAGTCAGGGTTATCGCTGTG-3′). Gene expression was normalized to that of 18S rRNA and is represented as the ratio relative to the indicated reference samples. All primers were validated for linear amplification.

### Statistical analysis

The data were analysed using two-tailed unpaired Student’s *t* test with Welch’s correction in cases of unequal variance. P values < 0.05 were considered statistically significant. All statistical analysis was performed using GraphPad Prism software (GraphPad).

## Additional Information

**How to cite this article**: Ohta, T. *et al*. Crucial roles of XCR1-expressing dendritic cells and the XCR1-XCL1 chemokine axis in intestinal immune homeostasis. *Sci. Rep.* **6**, 23505; doi: 10.1038/srep23505 (2016).

## Supplementary Material

Supplementary Information
