# Supplementary material for: Crucial roles of XCR1-expressing dendritic cells and the XCR1-XCL1 chemokine axis in intestinal immune homeostasis
Source: Sci Rep. 2016 Mar 23;6:23505. doi: 10.1038/srep23505 (PMC4804307; doi:10.1038/srep23505)
Supplement: Supplementary Information [file srep23505-s1.pdf]

Supplementary Information

Crucial roles of XCR1-expressing dendritic cells and the XCR1-XCL1 chemokine axis in intestinal immune homeostasis

Tomokazu Ohta[1,2,3], Masanaka Sugiyama[1,3,4,5], Hiroaki Hemmi[1,2,4,5], Chihiro Yamazaki[5,6], Soichiro Okura[1,2], Izumi Sasaki[1,2,5], Yuri Fukuda[1,2,5], Takashi Orimo[1,2,3], Ken J. Ishii[7,8], Katsuaki Hoshino[1,4,5,9], Florent Ginhoux[10] and Tsuneyasu Kaisho[1,2,4,5,*]

[1]Laboratory for Immune Regulation, World Premier International Research Center Initiative, Immunology Frontier Research Center, Osaka University, Suita, Osaka 565-0871, Japan

[2]Department of Immunology, Institute of Advanced Medicine, Wakayama Medical University, Wakayama, Wakayama, 641-8509, Japan

[3]Laboratory of Immune Regulation, Department of Microbiology and Immunology, Graduate School of Medicine, Osaka University, Suita, Osaka 565-0871, Japan

[4]Laboratory for Inflammatory Regulation and [5]Laboratory for Host Defence, RIKEN Center for Integrative Medical Science (IMS-RCAI), Yokohama, Kanagawa 230-0045, Japan

[6]Department of Immunology, Graduate School of Medicine, Dentistry, and Pharmaceutical Sciences, Okayama University, Okayama, Okayama 700-8558, Japan

[7]Laboratory of Adjuvant Innovation, National Institutes of Biomedical Innovation, Health and Nutrition, Ibaraki, Osaka 567-0085, Japan

[8]Laboratory of Vaccine Science, World Premier International Research Center Initiative, Immunology Frontier Research Center, Osaka University, Suita, Osaka 565-0871, Japan

[9]Department of Immunology, Faculty of Medicine, Kagawa University, Kita-gun, Kagawa 761-0793, Japan

[10]Singapore Immunology Network (SIgN), Agency for Science, Technology and Research (A*STAR), 138648, Singapore

*Corresponding author: Tsuneyasu Kaisho

Department of Immunology, Institute of Advanced Medicine, Wakayama Medical University, Wakayama, Wakayama, 641-8509, Japan

Tel: +81-73-441-0606 Fax: +81-73-445-5585

Email: tkaisho@wakayama-med.ac.jp

Supplementary Fig. 1 Generation of XCR1-DTA mice.

(**A**) Schematic representation of knocking the cre recombinase gene (cre) into the *Xcr1* locus. The mouse *Xcr1* wildtype allele, a targeting vector, and a cre recombinase knocked-in allele are shown. Filled and open boxes denote coding and non-coding regions of *Xcr1*, respectively. A neomycin resistance gene is flanked by yeast FRT sequences (frt). (**B**) Southern blot analysis of genomic DNAs from *Xcr1*$^{+/+}$ (+/+) and *Xcr1*$^{+/cre}$ (+/cre) mice. Genomic DNAs were isolated from mouse tails, digested with *Hinc*II and *Eco*RV, electrophoresed, and hybridized with a radiolabeled probe as indicated in (**A**). (**C**) Flow cytometry dot plots showing abundance of splenic, MLN and LP DCs in tissues from control (*Xcr1*$^{+/cre}$) and XCR1-DTA mice. XCR1-DTA mice were generated by crossing *Xcr1*$^{+/cre}$ mice with R26:lacZbpA^{flox}DTA mice. (**D**) Flow cytometry dot plots and histograms of splenic DCs from *Xcr1*$^{+/venus}$ mice are shown. Results are representative of five independent experiments.

Supplementary Fig. 2 Abundance of thymic, splenic, and MLN T lineage cells in XCR1-DTA and control mice.

(**A** and **B**) Percentages (**A**) and numbers (**B**) of thymic, splenic, and MLN T lineage cells in tissues from control (*Xcr1*$^{+/cre}$) and XCR1-DTA mice. Means ± s.e.m. of four mice are indicated (**B**). Results are representative of five independent experiments.

Supplementary Fig. 3 Intestinal T cell proliferation in XCR1-DTA and control mice.

(**A** and **B**) Histograms of T lineage cells of LP (**A**) and IELs (**B**) from control (*Xcr1*$^{+/cre}$) and XCR1-DTA mice, showing percentages of cells expressing the proliferation marker Ki67. Results are representative of two independent experiments.

Supplementary Fig. 4 Abundance and phenotype of Treg cells in XCR1-DTA and control mice.

(**A-C**) Percentages (**A**), intensity of Nrp1 expression (**B**) and numbers (**C**) of thymic, splenic, MLN, and LP Treg cells in tissues from control and XCR1-DTA mice. Shaded histograms and open histograms with red lines represent the data from control and XCR1-DTA mice, respectively. Open histograms with thin lines indicate labeling intensity with isotype-matched control antibodies. Means ± s.e.m. of four mice are indicated (**C**). Results are representative of four (**A** and **C**) or three (**B**) independent experiments. (*, $P<0.05$, Student's *t* test)

Supplementary Fig. 5   Generation of XCL1-deficient mice.

(**A**) Schematic representation of *Xcl1* targeting. The mouse *Xcl1* wildtype allele, a targeting vector, mutated and deleted alleles are shown. Filled and open boxes denote coding and non-coding regions of *Xcl1*, respectively. A neomycin resistance gene is flanked by yeast FRT sequences (frt). Mice with the mutated allele were crossed with CAG-*cre* transgenic mice to generate the deleted allele. (**B**) Southern blot analysis of genomic DNAs from *Xcl1* wildtype (+/+), heterozygous deleted (+/-), homozygous deleted (-/-) and heterozygous mutated (+/mut) mice. Genomic DNAs were isolated from mouse tails, digested with *Bam*HI or *Psy*I, electrophoresed, and hybridized with a radiolabeled probe as indicated in (**A**).

Supplementary Fig. 6 Splenic and MLN T lineage cells from control, XCL1- and XCR1- deficient mice.

(**A** and **B**) Percentages (**A**) and numbers (**B**) of splenic and MLN T lineage cells from control (*Xcl1*$^{+/-}$) and XCL1-deficient (*Xcl1*$^{-/-}$) mice are shown. (**C** and **D**) Percentages (**C**) and numbers (**D**) of splenic and MLN T lineage cells in tissues from control (*Xcr1*$^{+/venus}$) and XCR1-deficient (*Xcr1*$^{venus/venus}$) mice are shown. Means ± s.e.m. of five mice are indicated (**B** and **D**). Results are representative of four independent experiments. Statistical analysis was performed using Student's *t* test.

Supplementary Fig. 7 Abundance of splenic DC subsets in control, XCL1- and XCR1- deficient mice.

Percentages (**A** and **C**) and numbers (**B** and **D**) of splenic DC subsets from control (*Xcl1*$^{+/-}$) and XCL1-deficient (*Xcl1*$^{-/-}$) mice (**A, B**) and control (*Xcr1*$^{+/venus}$) and XCR1-deficient (*Xcr1*$^{venus/venus}$) mice (**C,D**) are shown. Means ± s.e.m. of five mice are indicated (**B** and **D**). Results are representative of four independent experiments. Statistical analysis was performed using Student's *t* test.

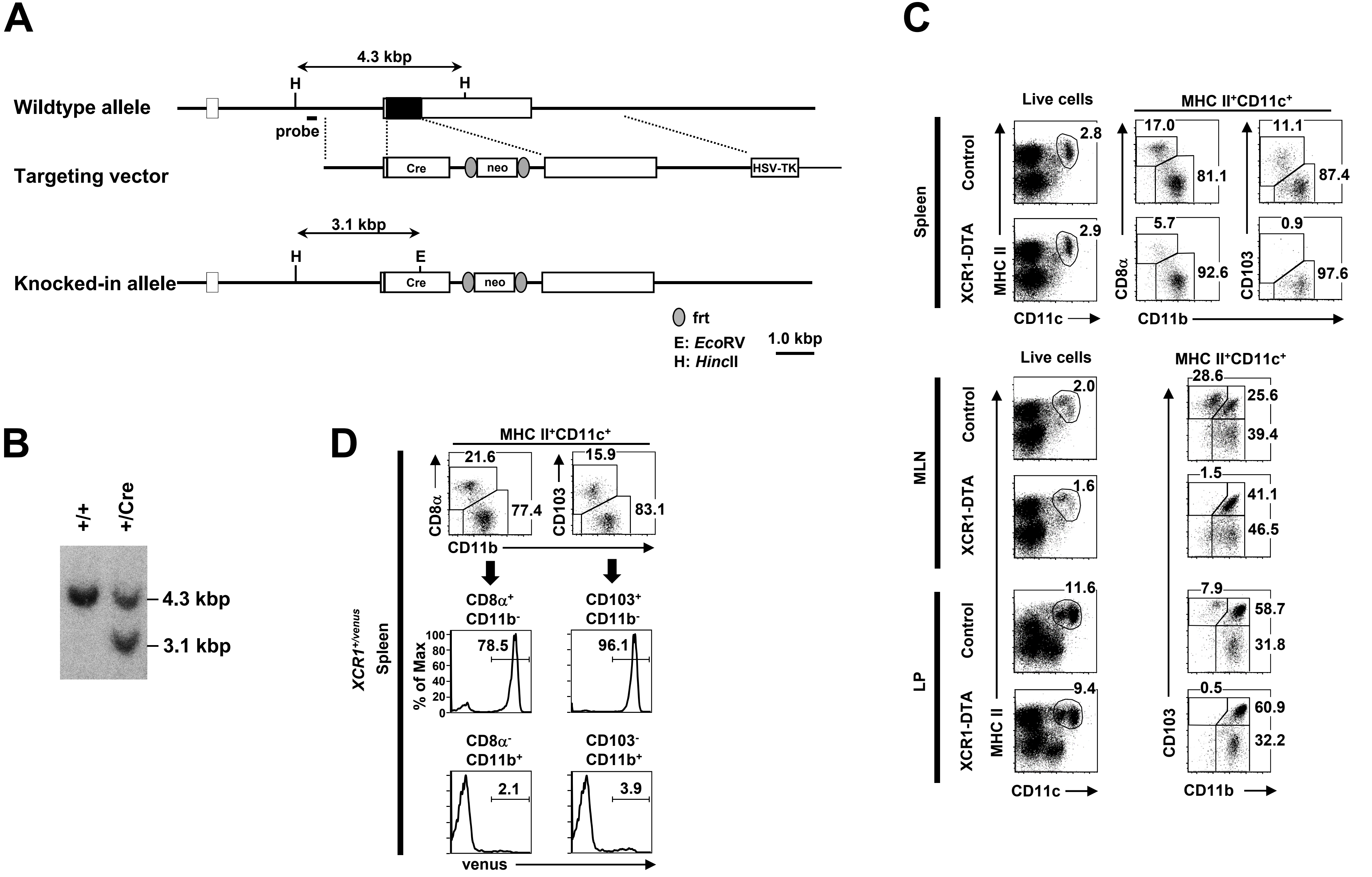

Supplementary Figure 1

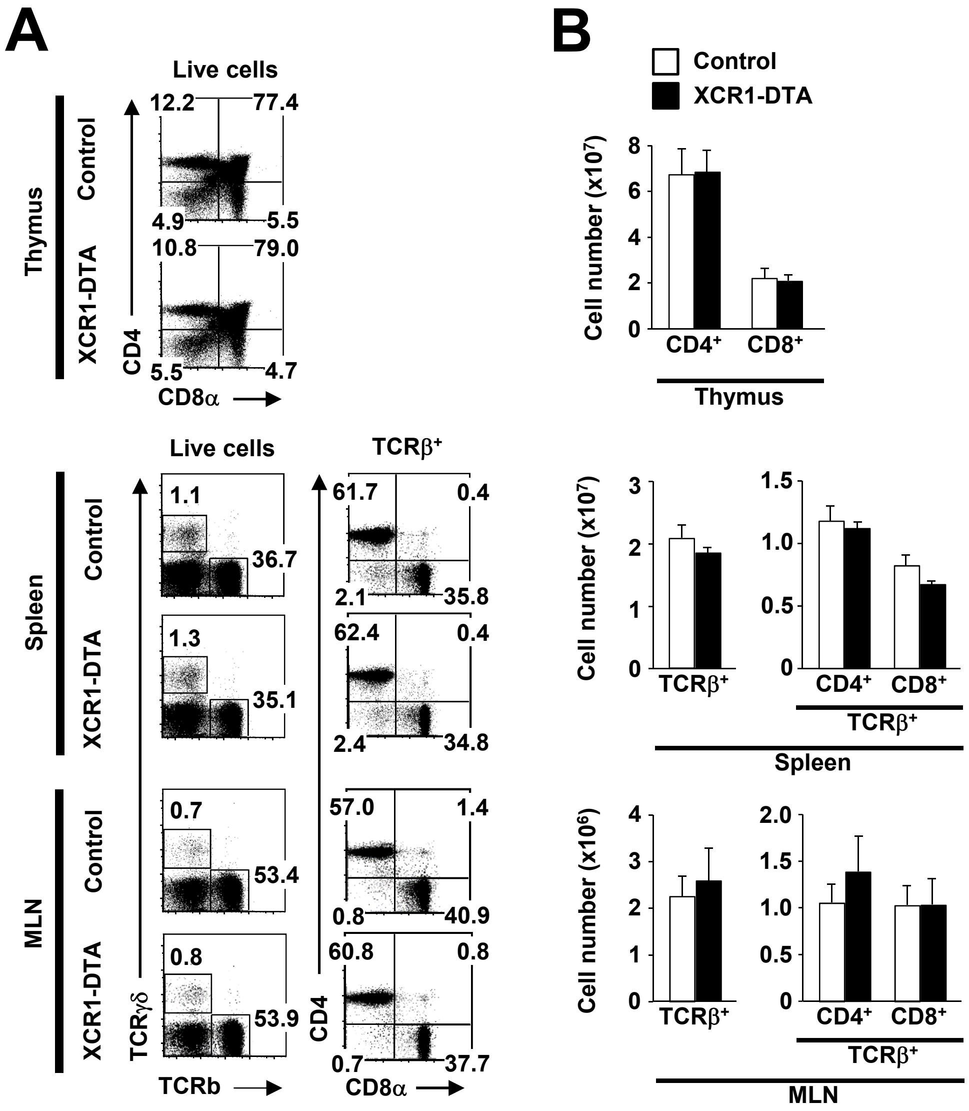

Supplementary Figure 2

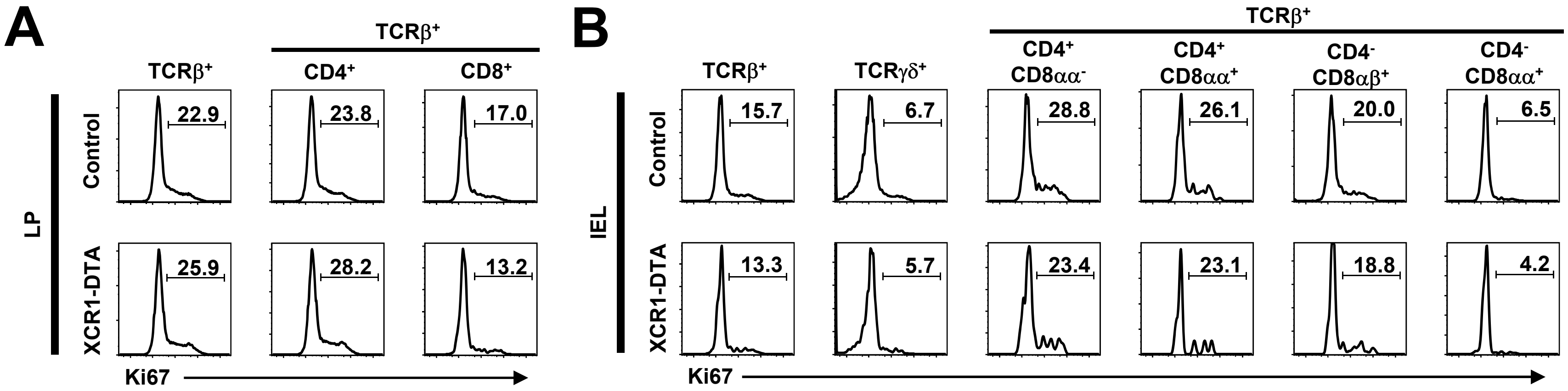

Supplementary Figure 3

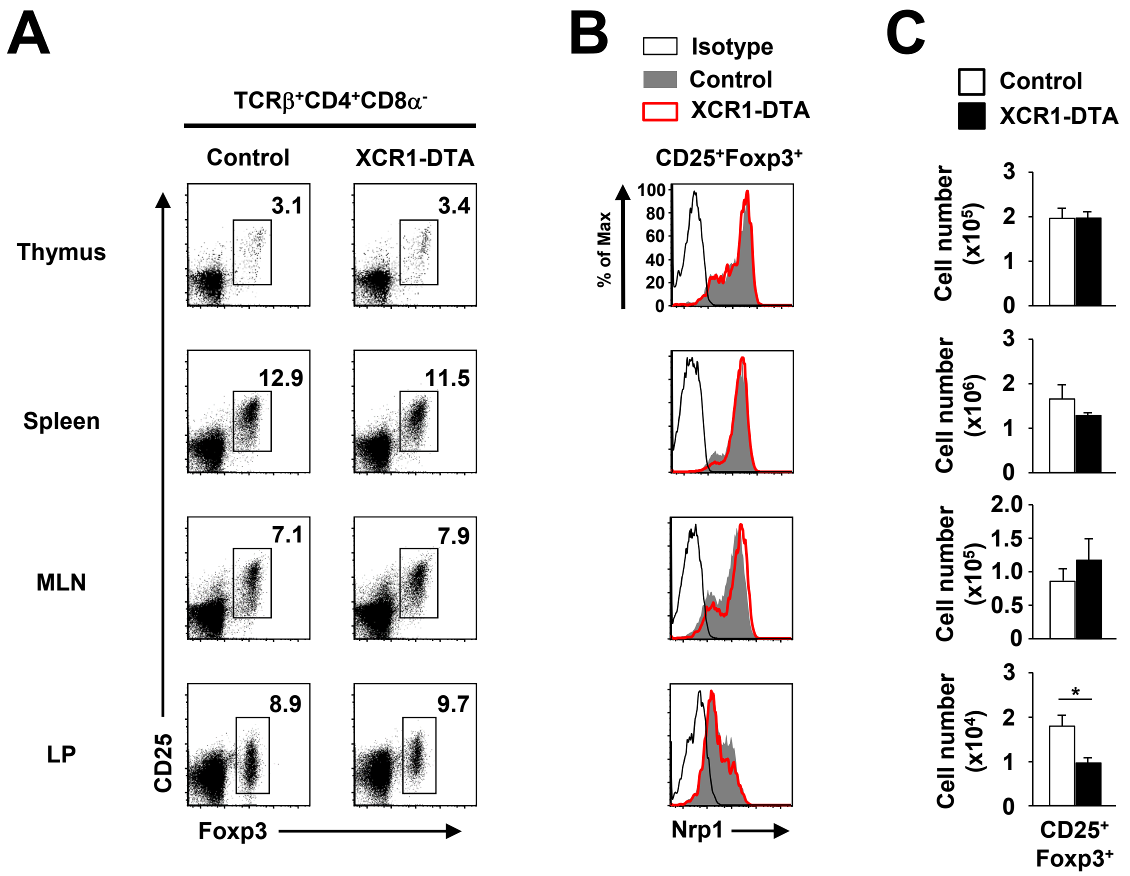

Supplementary Figure 4

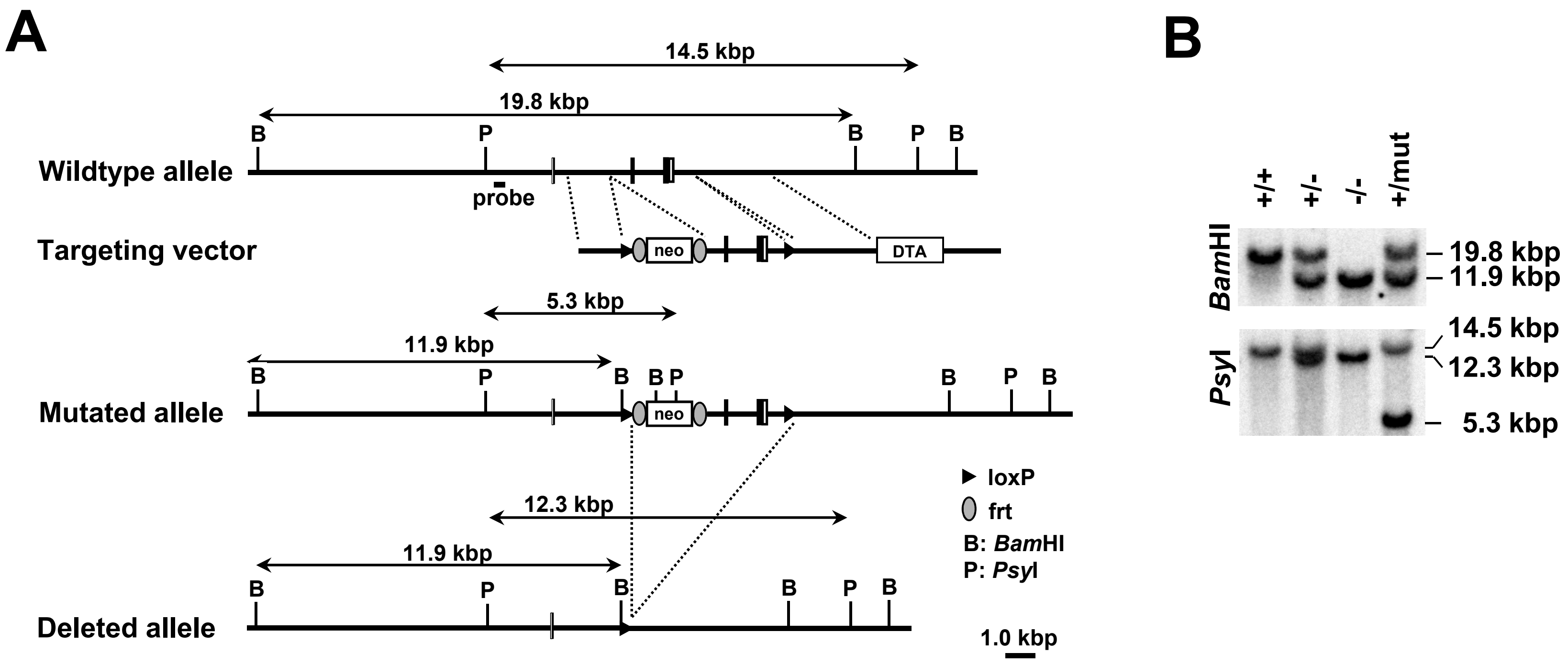

Supplementary Figure 5

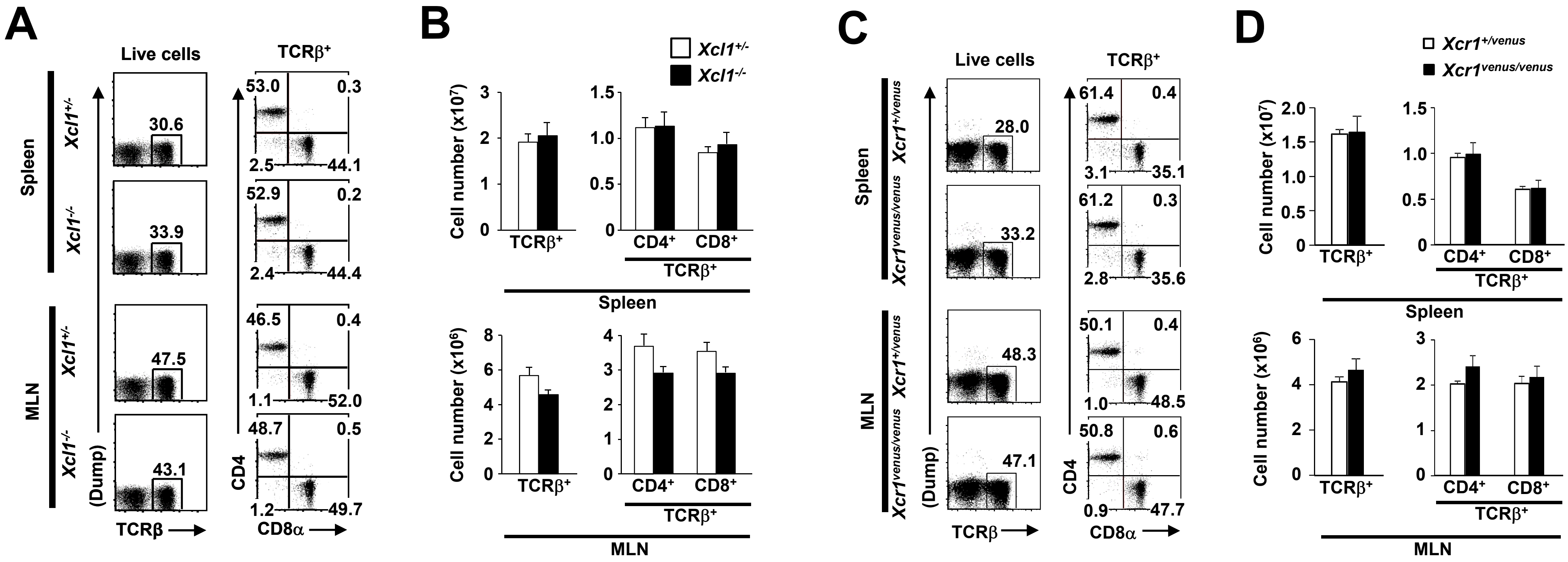

Supplementary Figure 6

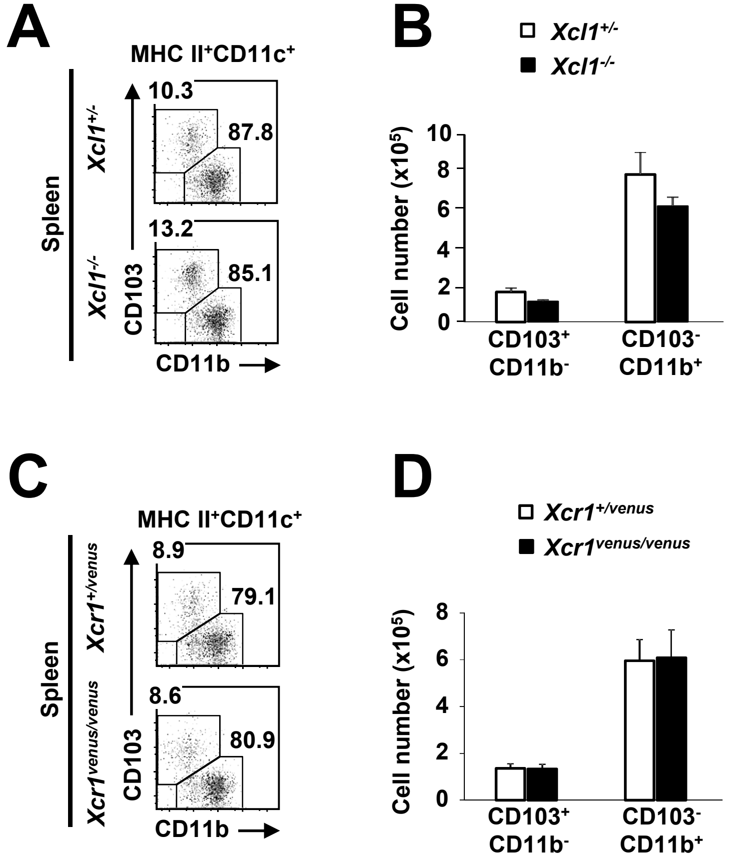

Supplementary Figure 7
